# Electrical impedance tomography as a tool for phenotyping plant roots

**DOI:** 10.1186/s13007-019-0438-4

**Published:** 2019-05-21

**Authors:** Diego D. J. Corona-Lopez, Sarah Sommer, Stephen A. Rolfe, Frank Podd, Bruce D. Grieve

**Affiliations:** 1 e-Agri Sensors Centre, The University of Manchester, Oxford Road, Manchester, M13 9PL UK; 2 Department of Animal and Plant Sciences, University of Sheffield, Sheffield, S10 2TN UK; 3 The University of Manchester, Oxford Road, Manchester, M13 9PL UK

**Keywords:** Root development, Plant pathogen detection, Electrical impedance tomography, *Plasmodiophora brassicae*, *Brassica napus* L.

## Abstract

**Background:**

Plant roots are complex, three-dimensional structures that play a central role in anchorage, water and nutrient acquisition, storage and interaction with rhizosphere microbes. Studying the development of the plant root system architecture is inherently difficult as soil is not a transparent medium.

**Results:**

This study uses electrical impedance tomography (EIT) to visualise oilseed rape root development in horticultural compost. The development of healthy, control plants and those infected with the gall-forming pathogen, *Plasmodiophora brassicae*—the causative agent of clubroot disease—were compared. EIT measurements were used to quantify the development of the root system and distinguish between control and infected plants at the onset of gall formation, approximately 20 days after inoculation. Although clear and stark differences between healthy and infected plants were obtained by careful (and hence laborious) packing of the growth medium in layers within the pots; clubroot identification is still possible without a laborious vessel filling protocol.

**Conclusions:**

These results demonstrate the utility of EIT as a low-cost, non-invasive, non-destructive method for characterising root system architecture and plant-pathogen interactions in opaque growth media. As such it offers advantages over other root characterisation techniques and has the potential to act as a low-cost tool for plant phenotyping.

**Electronic supplementary material:**

The online version of this article (10.1186/s13007-019-0438-4) contains supplementary material, which is available to authorized users.

## Background

Plant roots are complex structures that explore the soil, serving numerous adaptive purposes. The development of the root system architecture (RSA) is governed by both developmental (intrinsic) and environmental (extrinsic) signals, forming a three-dimensional underground network that provides anchorage for the above-ground plant body [1]. Other important root functions include the absorption of water and inorganic nutrients, storage of photosynthates and vegetative reproduction. The interaction between roots and the surrounding soil is highly dynamic. Root function changes soil properties, by both physical and chemical processes, releasing exudates into the rhizosphere and acting as an interface with microbial communities [2]. Plant–microbe interactions may be beneficial (e.g. mycorrhizae, rhizobia, plant growth-promoting rhizobacteria) or pathogenic (e.g. pathogenic nematodes, fungi, oomycetes, bacteria and Rhizaria such as *Plasmodiophora brassicae*—the causative agent of clubroot disease) [3].

Roots are therefore important targets for plant breeders, particularly with respect to responses to biotic and abiotic stresses [4]. However, visualising the RSA and associated root functions in plants grown in natural substrates is inherently difficult due to the opaqueness of soil, which has impacted breeding efforts. Numerous approaches have been developed for investigating RSA ranging from invasive (ex situ) to non-invasive (in situ) methods, including high-throughput root phenomics (reviewed in [4]). The simplest approach is excavation of field-grown plants with subsequent root washing and scoring of phenotypes. Such ‘shovelomic’ methods provide insight into plant roots grown in natural environments [5] but much of the 3D structure of the RSA is disrupted, only a snapshot view of the plant is provided and the technique is labour intensive. Roots growing in soil can be measured non-invasively and non-destructively using rhizotrons with transparent walls. However, rhizotrons provide limited access to the entire root system (minirhizotrons) or constrain root growth to 2D systems [6]. Alternatively, roots can be visualised using hydroponics or transparent substrates such as gellan gum or artificial soils [7, 8]. Such methods greatly facilitate visualisation and hence measurement of the RSA but may compromise physicochemical and microbiological features of the growth medium that are known to be important in root function.

Great advances have been made in using sophisticated tomographic imaging techniques to visualise the RSA of soil-grown plants including magnetic resonance imaging (MRI) and high-resolution X-ray computed tomography (µCT) [9–11]. Such methods allow 3D models of the RSA to be constructed with exquisite resolution but are expensive, require plants to be delivered to the imaging system and can require extended imaging times, limiting throughput and their adoption by the wider scientific community. In this study, we explore the use of electrical impedance tomography (EIT) as a non-invasive, non-destructive method for continuous monitoring of root development and the detection of below-ground plant diseases. This technique is low-cost, rapid and allows plants to be grown in horticultural composts with each pot equipped with measurement electrodes. Thus, EIT is amenable to high-throughput phenomics experiments.

### Electrical impedance studies in plant biology

An alternative to traditional root-study methods are technologies based on the concept that every biological subject has a defined electrical response. Electrical impedance (EI) techniques have been used to study plant properties such as root surface area [12], temperature tolerance [13], physical deterioration and plant growth status [14]. Electrical impedance spectroscopy (EIS) studies of roots have relied heavily on analogous electrical circuit modelling [15]. Typically, an alternate current of varying frequency is introduced through electrodes inserted in the stem and growth-medium [15]. However, there is no single electrical model to represent all RSAs’ bioelectrical responses due to the complexity of the system—the bioelectrical properties differ due to the electrode-medium interface, water content, stem and root dimensions, tissue density, growth medium, electrode-stem interface, electrode material and instrumentation. Nonetheless, EIS studies have been able to relate plant bio-electrical responses to root physiological effects [15]. The reliability of EI methods and its best implementations has been discussed thoroughly [12, 15, 16]; the authors show the importance of appropriate electrode configuration (3–4 terminal measurements achieve better results), the role of the stem-electrode contact when using needles or clamps, consideration of soil water content, and that application in field trials requires calibration against additional variables like mycorrhizas. Furthermore, to fully understand which frequency ranges and electrical properties offer more information about the RSA, methodological optimizations are continuously being discussed [17].

Imaging of soil electrical properties through Electrical Resistance Tomography (ERT) is a popular approach in agriculture and environmental studies using both 2D and 3D modalities. ERT is a low-intrusion, non-ionising, low-cost technique that provides both spatial and temporal subsoil data. Although ERT is commonly employed in Geophysics to image subsurface structures, other research areas are exploring the utility of this technique. Agriculture and plant-soil science have used ERT to determine factors such as soil composition, moisture content and salinity. These variables have been related to crop productivity [18], soil compaction [19], water content and flow in soil [20], soil cracks [21], tillage effects [22], moisture content in the root zone [20, 23, 24], water percolation [25], soil contamination [26], water uptake [27–29], and root biomass [30, 31]. Most of these studies have been conducted using trees with associated high root biomass; herbaceous plants are more challenging, as their smaller root biomass makes it difficult to distinguish between roots and changes in soil moisture [23]. Thus, a key challenge for ERT is the variability of soil conditions (e.g. soil texture, compaction, particle distribution, porosity), as this can lead to ambiguities when trying to explain the results obtained. These ambiguities are accentuated when the RSA impact on the growth-medium is ignored. As explained before, it is difficult to identify the individual contribution of each root segment to bioelectrical measurements due to a high number of variables. Attempts to overcome some of these problems were made recently through the use of ERT with a mise-à-la-masse approach [27], ERT in conjunction with electromagnetic inductance imaging [32], time-lapse ERT and independent measurements with a field-scale calibration method using soil thermal profiles [33]. Furthermore, Cassiani et al. [34] and Vanella et al. [35] demonstrated how ERT in conjunction with other technologies, soil moisture sensors and eddy covariance systems respectively, can be used to characterise the spatial distribution of water content and nutrient uptake. While it might not be possible to detect individual root interactions through this technique, ERT can detect root related processes.

Electrical impedance tomography (EIT) shares the same concepts as ERT. Although the imaged property remains the same (resistivity, or its inverse conductivity), EIT injects an alternating current rather than a direct current. This provides extra versatility as it can exploit different modalities (time difference, frequency difference, multi-frequency) and expands the opportunities to characterise plant-soil interactions. The conduction of current in RSAs depends highly on the characteristics of the AC injected with the potential to better discriminate between soil and root tissues. Recent advances in this area [28, 36], show that the use of spectral EIT in combination with induced polarisation techniques are capable of discerning the presence of high-density roots and some physiological processes. Moreover, studies such as [37], show the capability of EIT to identify the presence of pathogenic agents in trees. This field is still under continuous development, and new approaches are being established. For instance, Rao et al. [38] suggest a combined use of ERT and plant-water-flow models to further understand their pedophysical interactions with soil.

An EI measurement is obtained from the electrical response (voltage) to an applied electrical excitation (current). An EI measurement of a pot containing a growing plant root will depend upon the different electrical properties of the root and constituents of the growth substrate, the moisture content and ionic strength. Thus, the root will physically alter the overall impedance of the system as it grows, compacts and displaces the growth medium as well as resulting in ionic changes due to water and solute uptake and root exudate production. Therefore, impedance is considered as a composite measure of the physical and physiological processes within the pot. The main limitation is that the spatial resolution of the EIT system is much lower than that of X-ray and nuclear magnetic resonance (NMR) based methods, and as such, should be considered complementary to these techniques. Here we use EIT to examine oilseed root development in compost filled pots and the impact of the gall-forming disease, Plasmodiophora brassicae (clubroot) on the signals and images obtained.

### EIT and clubroot

Clubroot was chosen to test the utility of EIT as a tool for plant health diagnosis and detection of below-ground plant diseases. *P. brassicae* is a eukaryotic biotrophic pathogen of vegetables and crop plants within the Brassicaceae family throughout the temperate regions of the world including the UK [39]. Infected plants develop galls in the root system that reduce yield by acting as a sink for carbohydrate and disrupting water and nutrient uptake [40]. These symptoms result from perturbation of host metabolism, stem cell maintenance and differentiation, and vascular development [41]. Control of the disease is problematic. Chemical controls are limited and expensive, while complete sanitisation of field machinery, required to stop the disease spreading, is difficult. Breeding resistant plants is therefore preferential, but the existing monogenic resistance has already been broken down in the field due to selection pressure and genetic variability of the pathogen [40]. This has led to outbreaks of clubroot throughout the world. There is therefore an urgent need to find new sources of clubroot resistance genes (both quantitative and qualitative). This endeavour would be greatly facilitated by accurate root phenomic methods to quantify disease development.

In this manuscript, we describe how EIT can be used to visualise the developing root system of oilseed rape plants in compost-filled containers and the results used to extract parameters that reliably detect the impact of *P. brassicae* infection at the onset of gall formation. These measurements can be made on a daily basis providing a novel route to explore root development and response to disease.

## Results

Control (healthy) and *P. brassicae*-infected oilseed rape plants (variety Temple) were grown in horticultural compost in EIT chambers. The chambers were filled with compost, initially using a complex, and time-consuming, packing protocol and later a simplified packing protocol. A summary of the experiments performed is provided in Table 1. 3D reconstructions of changes in conductivity were calculated on a daily basis. To visualise the developing root system and associated changes in soil conductivity, reconstructions were performed using the normalised difference against a reference data set (the same pot before prior to transplantation of a seedling) (as discussed in “Methods”). Thus, the results represent the normalised change in conductivity (Δσ) against a reference. Representative reconstructions are presented as 3D iso-volumes and 2D slices at specific positions. Graphs of the average Δσ and the standard deviation (SD) for the entire Volume of Interest (VOI) (0–13 cm), upper (6.5–13 cm) and lower (6.5–0 cm) regions of all pots are shown. Due to equipment limitations, separate experiments were performed: compost only (Additional file 1: Figure S1), control plants, infected plants (1) and infected plants (2). Experiments were performed keeping the compost at field water capacity throughout.

**Table 1:** Experiments carried out in this investigation using *Brassica napus L.* (variety Temple)

Experiment	No. pots	Duration	Compost bulk density	Inoculation	Compost packing protocol
Compost only	3	31 days	105 mg/cm^3^	None	Complex
Control plants	6	35 days	80 mg/cm^3^	None	Complex
Infected plants (1)	6	35 days	80 mg/cm^3^	*P. brassicae*, 50 ml of 6.25 × 10^5^ spores/ml	Complex
Infected plants (2)	6	31 days	90/105 mg/cm^3^	*P. brassicae*, 50 ml of 6.25 × 10^5^ spores/ml	Simple

**Fig. 1:**
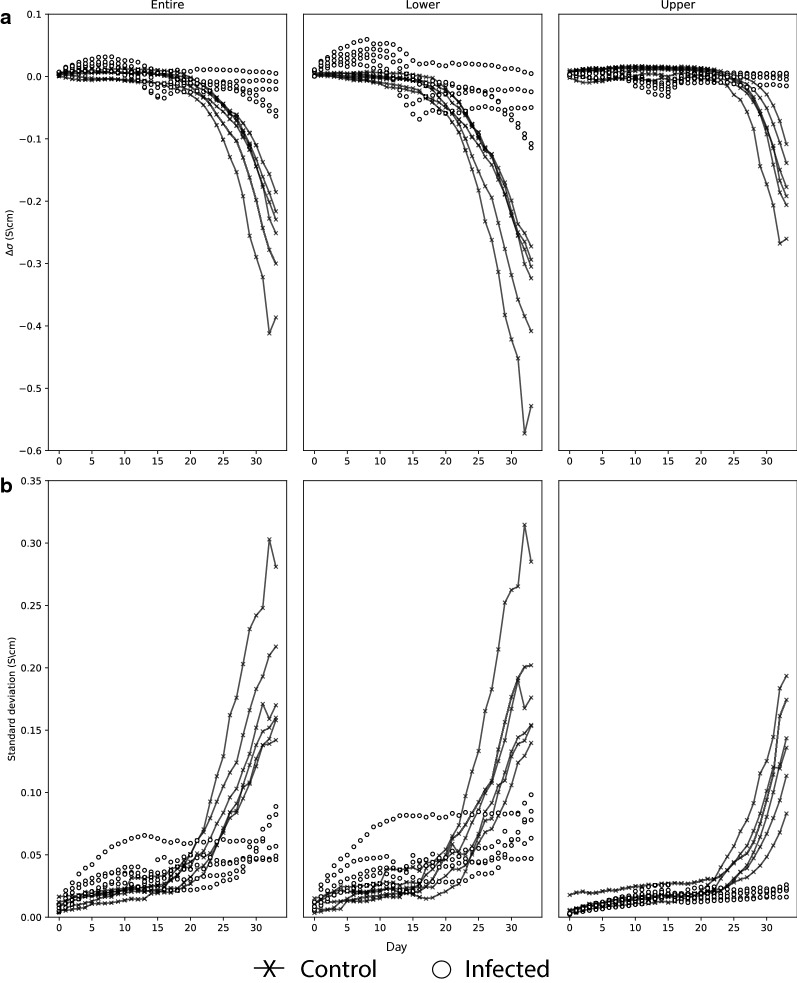
Conductivity changes across volume of interests for control and clubroot-infected plants. Measurements were made daily after inoculation. Average values across the VOI (**a**) and its standard deviation (**b**) within the VOI are shown for the upper (6.5–13 cm), lower (0–6.5 cm) and the entire (0–13 cm) pot. Results are normalised to Day 0 for each replicate

Figure 1 shows the daily results for both control and infection (1) experiments using an 80 mg/cm^3^ bulk density. In control experiments, the predominant change observed was a decrease in conductivity (or resistivity increase) and an associated increase in heterogeneity (standard deviation of the conductivity—SD). The Δσ of the entire VOI remained relatively constant until the 17th day, with only small fluctuations of the order 10^−3^, but then conductivity decreased sharply reaching values between − 0.2 and − 0.4 S/cm by the end of the experiment. The SD increased sharply at this time from values > 0.05 S/cm to values reaching 0.3 S/cm. Dividing the VOI into upper and lower regions showed that the greatest changes in conductivity and SD occurred in the lower region, with a marked decline in conductivity from 15 days onwards reaching values between − 0.3 and − 0.6 S/cm by the end of the experiment. In the upper region, changes were smaller (between − 0.1 and − 0.2 S/cm) and started later (23 days onwards). These marked changes in conductivity and SD were only seen in pots containing growing plants; in pots containing compost only, the average change in conductivity observed after 30 days was only 2 mS/cm (Additional file 1: Figure S1). Finally, the application of an analysis of variance (ANOVA) to the EIT dataset indicated no significant differences between control plants (p > 0.05).

In experiments with clubroot-infected plants, changes in Δσ and SD were slower and less extensive. In 3 replicates, no significant difference in Δσ and SD was evident at the end of the experiment (30 days post inoculation) compared to initial values, whilst in two replicates Δσ at the end of the experiment in the entire VOI was approximately − 0.05 S/cm with little change in SD. Again, the greatest changes were evident in the lower VOI. Two replicates showed a reduction in Δσ to − 0.133 S/cm but there was no change evident in the upper VOI. ANOVA found there was a statistically significant difference between control and infected samples (p < 0.001) evident from 23 dpi onwards for the entire VOI and 21 dpi in the lower VOI. Values in the upper part of the pot were more strongly influenced by watering regimes and were variable.

**Fig. 2:**
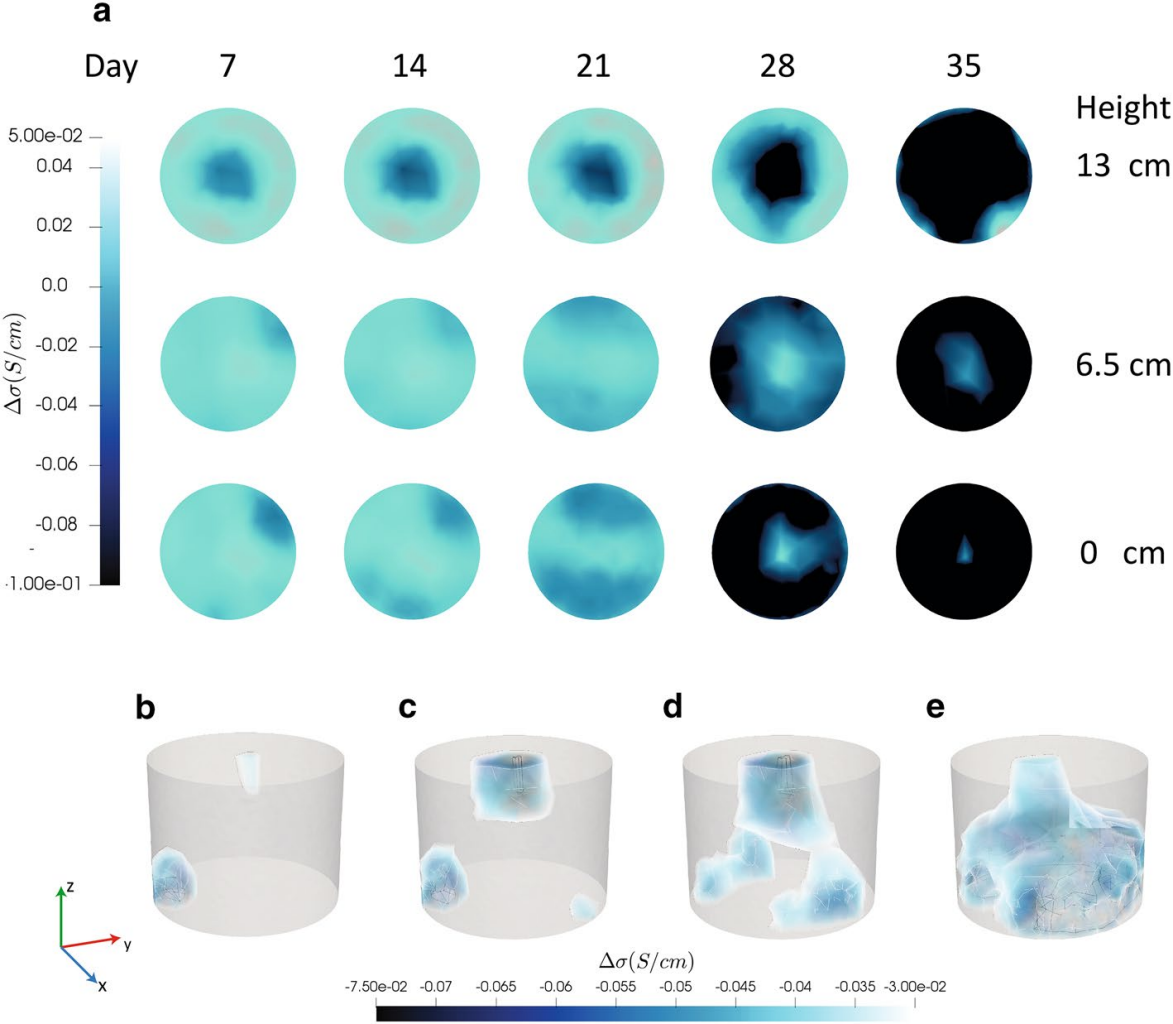
Reconstructions of conductivity in a representative control *B. napus* plant. **a** Cross-sections of conductivity changes in the top (13.5 cm), middle (6.5 cm) and bottom (0 cm) of the pot at selected timepoints. 3D iso-volumes with range − 75/− 30 mS/cm are shown at 2 (**b**), 10 (**c**), 20 (**d**) and 25 (**e**) days. The XYZ scale is centimetres

**Fig. 3:**
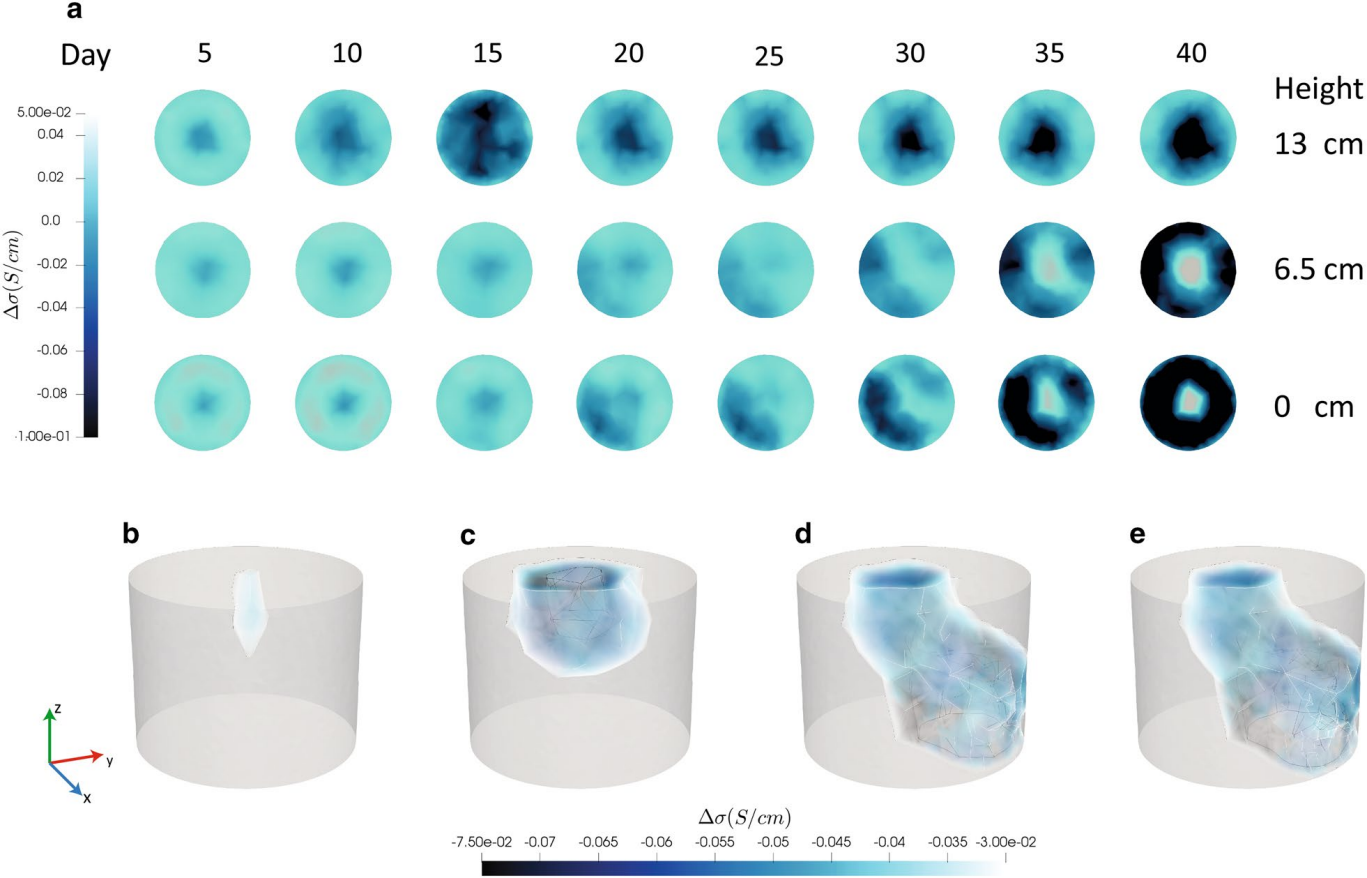
Reconstructions of conductivity in a representative clubroot-infected *B. napus* plant. **a** Cross-sections of conductivity changes in the top (13.5 cm), middle (6.5 cm) and bottom (0 cm) of the pot at selected timepoints. 3D iso-volumes with range − 75/− 30 mS/cm are shown at 2 (**b**), 10 (**c**), 20 (**d**) and 25 (**e**) days. The XYZ scale is in centimetres

2D cross-sections and 3D iso-volumes of control and clubroot-infected plants at selected time points are shown in Figs. 2 and 3 respectively. The range of the 3D iso-volumes corresponds to the most negative value found at the top centre of the vessel on the 5th day, and 40% of that value (i.e. from − 75 to − 30 mS/cm).

Conductivity images of pots containing control plants showed a central intrusion in the upper region of the pot (Fig. 2, day 7). The diameter of the extrusion expanded as the experiment progressed. In the middle and lower VOIs of the pot, more extensive changes in conductivity occurred as the experiment progressed until a uniform reduction in conductivity across the whole region was observed. By the end of the experiment, the EIT chamber was full of plant roots (Fig. 5c and Additional file 2: Figure S2A). 3D iso-volumes of chambers containing clubroot-infected plants showed that the region of altered conductivity was smaller and distorted compared to control plants. At the end of the experiment, plant roots were excavated and galls were present in all of the infected plants.

**Fig. 4:**
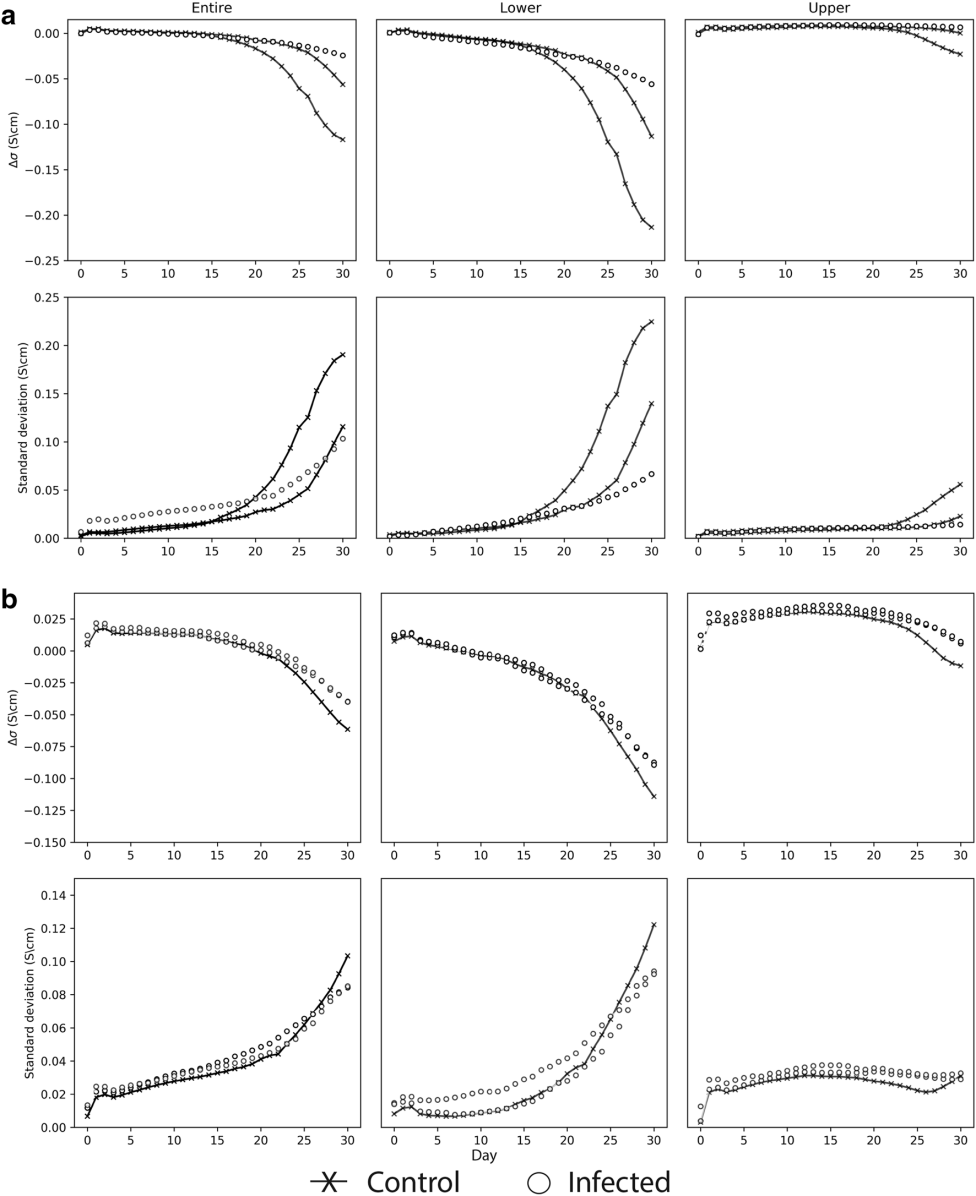
Conductivity changes for control and clubroot-infected plants at different soil packing densities. Measurements were made daily after inoculation for pots packed at 90 (Density 1) (**a**) and 105 (Density 2) (**b**) mg/cm^3^. Average values across the VOI and its standard deviation within the VOI are shown for the entire pot (0–13 cm), the upper section (6.5–13 cm) and lower section (0–6.5 cm) and the entire (0–13 cm) pot. Results are normalised to Day 0 for each replicate

These experiments were performed using a laborious protocol to fill the chambers with compost. As the application of EIT in monitoring root growth and development in a high-throughput environment would be greatly facilitated by avoiding the need for lengthy pot filling protocols, an experiment [Infection (2)] was performed where pots were simply filled with unsieved compost at two different packing densities (90 and 105 mg/cm^3^). Plants were mock-inoculated with water or with *P. brassicae* spores and EIT measurements were made daily for 31 days. Average conductivities and variation for the entire pot (All), upper and lower sections are shown in Fig. 4. At the lower packing density of 90 mg/cm^3,^ marked differences in the Δσ and SD of control and infected plants were evident from 17 dpi onwards, particularly in the lower VOI. In contrast, differences between control and infected plants were much smaller in chambers filled at the higher packing density of 105 mg/cm^3^. The overall changes in Δσ were also smaller, − 0.1 to − 0.20 in the lower VOI of chambers filled at 90 mg/cm^3^ compared with − 0.075 to − 0.1 in the lower VOI of chambers filled at 105 mg/cm^3^.

## Discussion

In this article we have demonstrated that EIT can provide non-invasive, non-destructive parameters of root development and distinguish between control and clubroot-infected plants at the onset of gall formation, which typically commences 20 days after inoculation in oil seed rape. The use of our growth-medium preparation protocol removes large particles of organic content. Thus, the medium shares the same constant electrical characteristics seen in Additional file 1: Figure S1. One of the major factors influencing the conductivity of the growth medium is water content—a decline in water content would increase soil pore air [42, 43], and lead to poor electrical contact between roots and growth-medium. Therefore, the vessels were kept at field water capacity during the course of the experiment and the medium behaved in a constant manner with minor changes only (as shown from the results in the Additional file 2: Figure S2) caused by transpiration, soil settlement, and instrumentation errors. These conditions are also appropriate for studying clubroot infection as development of the disease is favoured in wet soils [44].

Depending on specific growth medium characteristics (for instance, moisture, nutrient content, and salinity) and plant species, roots can exhibit higher electrical impedance than the medium where they grow [12, 13, 20, 21]. Our experiments employ a growth medium with a considerable amount of mobile ions and were carried out at field capacity; hence we hypothesise that medium disruption by the growing root system and associated changes in electrolytes (such as nutrient uptake and formation of depletion zones) [45] are the primary causes for conductivity decrease observed in the EIT systems. This is consistent with the timing of the changes in conductivity, greater changes in the lower VOI as the root system develops (filling the chamber by the end of the experiment) and impact of *P. brassicae* infection which inhibits RSA development as a consequence of gall formation [44].

Detection of individual roots is challenging [20, 21], mainly due to the limited resolution of EIT. Nonetheless, the presence of roots in soil does affect its electrical conductivity, thus, we can infer their position by their interactions. Consistent with [21], the changes in conductivity are more evident at higher root densities—if the root density is low, changes in conductivity are more difficult to differentiate between root interactions and the soil’s natural processes.

In the current study it was possible to obtain low resolution tomographic images of the root system, but the greatest utility of the approach results from parameters reflecting changes in conductivity from the starting condition and variation in conductivity within the system. The iso-volumes shown in the Results can be interpreted broadly as a convex hull of the main RSA, reflecting the natural development of *Brassica napus* L. RSA [46]. These simple parameters provide a fast and qualitative measure of disease development. At present, breeding for clubroot resistant varieties is based upon destructive sampling at single time-points and simple scoring methods [47] thus EIT will provide a novel approach for breeders.

The ability to obtain impedance parameters from discrete sub-volumes of the pot provides greater insight into the impact of treatments on the RSA. In this study, parameters were calculated for the entire pot, upper and lower regions. Analysis of variance in the lower region gave improved disease detection 2–3 days earlier than the entire pot, but values from the upper region were more strongly influenced by the effects of seedling transplantation, soil settling and watering. However, these perturbations were restricted to the first 5 days and did not significantly influence values obtained at later time-points. In future, more refined selection of informative volumes may improve further the ability to detect disease development. Results were affected by soil density but, after an initial settling period, laborious soil packing approaches were found not to be necessary to obtain useful results, greatly increasing the utility of the system. Nevertheless, we emphasize the importance of growth-medium preparation to obtain improved results. Depending on the complexity of the growth-medium chosen (e.g. type of soil, organic content, compaction, etc.) resolution of this technique could decrease. For instance, the presence of large organic particles could lead to lower/higher conductivity values in that area; moreover, if the reconstruction model does not consider the location of these particles, it could lead to false interpretations. Soil bulk density has an effect on root development [48] (it can affect root diameter, length, total biomass). While we did not fully explore the effects of bulk density, we acknowledge the importance of choosing a density that enhances root growth when using this technique. As mentioned before, higher root densities are easy to detect. In our case, this greatly improves the early detection of clubroot infected plants.

### Developments

As the EIT approach is entirely scalable in volumetric dimensions, the approach may be extended to image plant roots in pots of different sizes to those used in this study, accommodating differing root architectures. However, variations in spatial resolution and sensitivity exist within the volume to be imaged. For regularly-spaced arrays of circumferential electrodes, located at equal separation down the vertical axis of a cylindrical pot, these areas of low sensitivity tend to occur towards the centre line of the cylinder. This is due to the decrease in the effects of impedance variation that occur near the centre-line. The ill-posed nature of the soft-field inverse image reconstruction problem is such that the addition of further electrodes at the periphery has diminishing returns on increasing the spatial resolution. This limitation results in the resolution of the system being approximately 5% of the diameter of the vessel being imaged. This gives rise to a design compromise in electrode numbers per electrode plane, with 16 or 32 electrodes employed typically, versus the overall cost and complexity of the system. To mitigate for this, various alternate electrode geometries have been proposed and developed by research groups, including the addition of electrodes within the measured volume. The latter will affect the volume under investigation but could be an acceptable compromise in some phenotyping applications. Alternatively, or additionally, it is possible to reduce the ill-posed nature of the reconstruction, and deliver image data with increased spatial resolution, by incorporating prior information on the nature of the soil-crop system such as from fluid flow modelling [49].

Design compromises also exist in the selection of drive currents and frequencies, and the corresponding voltage amplitude and phase measurements. When using a sinusoidally modulated drive current with drier soil types and/or large soil particulates with high volume fraction, the lack of a continuous low-electrical resistance path through the medium dictates that the orthogonal out-of-phase reactive (capacitance) currents will dominate in the measured signals. This implies that a higher frequency of oscillation would be preferable to maximise the SNR under these conditions, due to the inverse relationship between angular frequency and capacitive conductivity. However, as such scenarios can result in femtofarad (10^−15^ F) measurements, parasitic capacitance between cabling within the system and circuitry tends to limit the useable operating frequencies to between 100 kHz and 1 MHz in most practicable situations. Larger electrode plates and careful electronics design using high dielectric materials may help to increase the useable upper frequency, but with corresponding effects on reduced spatial resolution or increased cost, respectively.

Similarly, the amplitude of the drive currents would ideally be increased for wet soils with high electrolyte compositions, i.e. low electrical resistance, so as to maximise the measured voltages. However, Ohmic heating limits the usable current levels, not just due to the potentially damaging effects of the temperature increase, but also due to the resulting current drawn from the system making it impracticable for microelectronic design approaches. Conversely, for wetter soils with lower concentrations of electrolytes, the mid-scale impedances of even modest currents can result in comparatively high driven electrode voltages and the formation of a double-layer capacitor as well as other parasitic effects at the electrode boundary [50]. As a result, future systems for soil-borne phenotyping may be developed with measurement protocols that autonomously modulate the frequency, currents and drive-receive electrode combinations to maximise SNR versus these effects, using either a combination of sinusoids or Fourier transform deconvolution of non-sinusoidal drive signals.

As mentioned above, challenges remain and further developments needed to improve EIT. EIT can be used as a quantitative technique as shown by [27] when used in conjunction with other techniques. Researchers have used ERT in conjunction with ground penetrating radar and electromagnetic induction to enhance the spatial resolution and quantitative abilities of these techniques [51]. In this study we made considerations to create a more natural environment for plant growth. Recent studies [28, 38] employ 2D rhizotrons that allow the direct observation of the RSA. The advantage of such systems is that the quantitative ability of EIT could be further explored by scanning root length with other computed-tomography methods. Nevertheless, our research shows that EIT can enable RSA study scenarios with less root development constraints (e.g. the shape of the containing vessel).

Although modifications to the current EIT instrumentation could be desirable, the current study has demonstrated the system’s utility in quantifying responses to the gall-forming disease *Plasmodiophora brassicae* and, with suitable multiplexing of measurement pots and development of microelectronics, could be deployed in screens disease phenomics screens that seek to identify quantitative resistance to this important disease. EIT could also be used to investigate other root pathogens such as *Rhizoctonia,* plant-parasitic nematodes or root herbivores, particularly in studies exploring host-rhizobiome-pathogen interactions which require the use of natural substrates [52, 53]. The sensitivity of EIT to changes in soil moisture will also allow breeding of plants with changes to RSA development and water use efficiency, particularly in longer pots that can capture the full depth of the root system that would be encountered in the field.

## Methods

### Plant growth and *P. brassicae* inoculation

*Brassica napus L.* (variety Temple) seeds were germinated on moist filter paper in darkness at 20 °C for 3 days. A seedling was then transferred to the centre of each EIT vessel (described below) filled with Levington Advance Seed & Modular F2 [ICL, (UK) Ltd]. This a peat-based substrate with a particle size of approximately 0–3 mm, 269–329 µs/cm electrical conductivity range, 5.3–6.0 pH range and added nutrients 144 N, 73P, 239 K. Plants were grown at an irradiance of 220 µmol/m^2^/s for 5 days to allow seedling establishment and then 330 µmol/m^2^/s with an 18 h photoperiod at 20 °C. Plant positions were randomised every 3 days. The pots were stood in 1 cm of water (changed every 3 days) with an additional 50 ml added from above the growth-medium every 2 days. For inoculation with *Plasmodiophora brassicae,* 50 ml of 6.25 × 10^5^ spores/ml suspension of clubroot spores was added immediately after transplantation. Spores suspensions were produced as described in [41].

### Growth-medium preparation

**Fig. 5:**
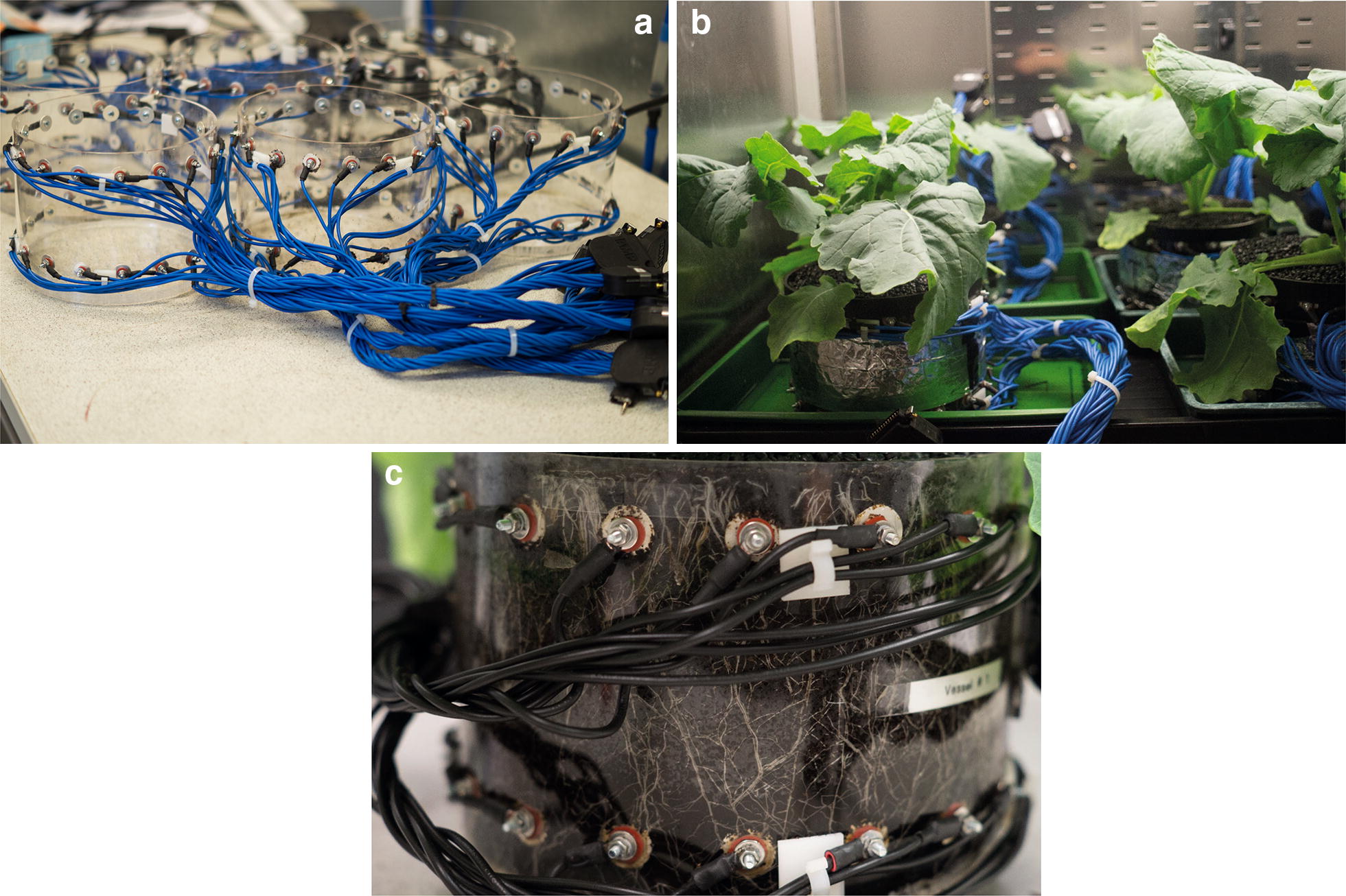
EIT equipment. **a** Empty EIT vessels showing the arrangement of the electrodes. **b** Vessels in the growth chamber. **c** Root growth in control plants at the end of the experiment

The EIT pots consisted of a Perspex cylinder 13 cm high with a diameter of 18 cm and two rings of 16 silver plated electrodes (radius 0.75 cm) positioned at z = 2 cm and z = 11 cm (Fig. 5).

**Fig. 6:**
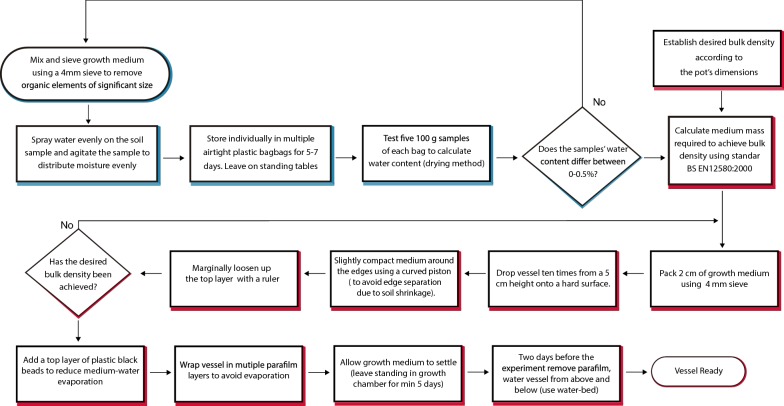
Growth-medium preparation and pot-filling protocols. Blue and red shadowed boxes correspond to preparation and pot-filling protocols respectively

Initial experiments used a bulk density of 80 mg/cm^3^. A growth medium preparation protocol was designed to overcome some of the limitations of using peat-based compost (e.g. difficult to moisten once dry). Figure 6 describes the growth-medium preparation and pot filling protocols followed in the experiments. The aim of the protocols is to maximize the homogeneity between samples, in order for them to start with a similar electrical conductivity. These experiments use a transparent vessel. For this reason, walls were wrapped in aluminium foil to prevent algal growth. The seedlings were transplanted in the vessel’s centre at 2 cm depth in a small incision made with small finger.

A second set of experiments used pots that were simply filled with unsieved compost (prepared using just the filling protocol) to determine whether EIT reconstructions could be achieved without the need for extensive soil homogenisation. Pots were filled at two bulk densities—90 and 105 mg/cm^3^. Table 1 summarises experiments’ conditions carried in this study.

### EIT instrumentation and image reconstruction

**Fig. 7:**
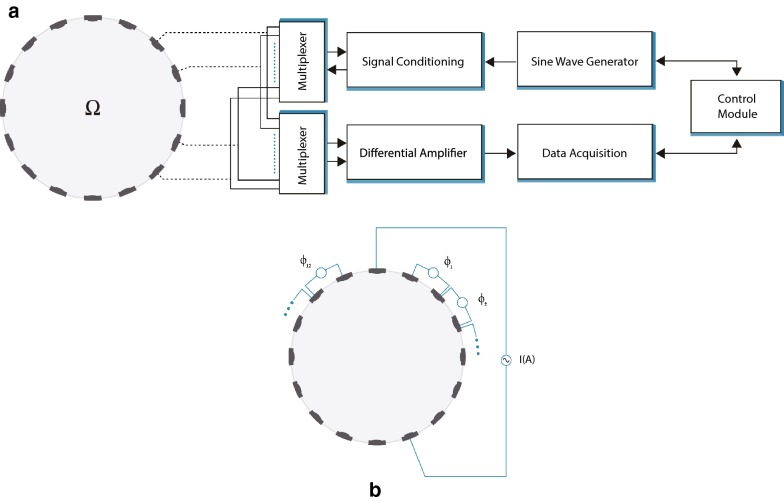
The EIT control system. **a** A schematic of the LCT control system. Voltage measurements are made between pairs of electrodes (φ_n_) with current I(A) injected into (**b**) opposite pairs of electrodes

EIT maps the distribution of conductivity within an object by measuring the electrical potential at the body’s boundaries. Signals were generated and analysed using the LCT2 (low-cost tomography system v2) developed at the University of Manchester in collaboration with Syngenta [54]. A 1 mA signal was injected into one pair of electrodes at a 5 kHz frequency as preliminary EIS experiments showed that this frequency provided the best compromise between achieving a linear relationship between injected and measured signal amplitudes, whilst also offering a suitable Signal-to-Noise (SNR) ratio to compute the phase angle [55]. The induced voltages were then measured between the other pairs of electrodes. This 4-electrode configuration (Fig. 7b [55]) helps minimise the effects of electrode contact electrochemical impedance (polarisation). Measurements were made in the middle of the night period. Signals may be injected using any combination of two or more electrodes, however to simplify the analysis, in this early investigation, only the opposite stimulation strategy was studied. A schematic of the system and the measurement strategy is shown in Fig. 7.

As the EIT pots contain two rings of 16 electrodes, 384 measurements are made with the opposite strategy. However, only truly independent measurements contribute to the final solution with singular value decomposition (SVD) analysis showing 184 independent pairs. The opposite strategy distributes current more evenly across the EIT vessel providing better SNR to changes in the centre of the system where the plant is placed. All reconstructions were obtain using reference data set measured before plants were introduced into the system (time-difference EIT). Moreover, we used a normalised difference, i.e. (Meas. − Meas. _ref_)/(Meas. _ref_), after solving the inverse problem for visualisation purposes.

**Fig. 8:**
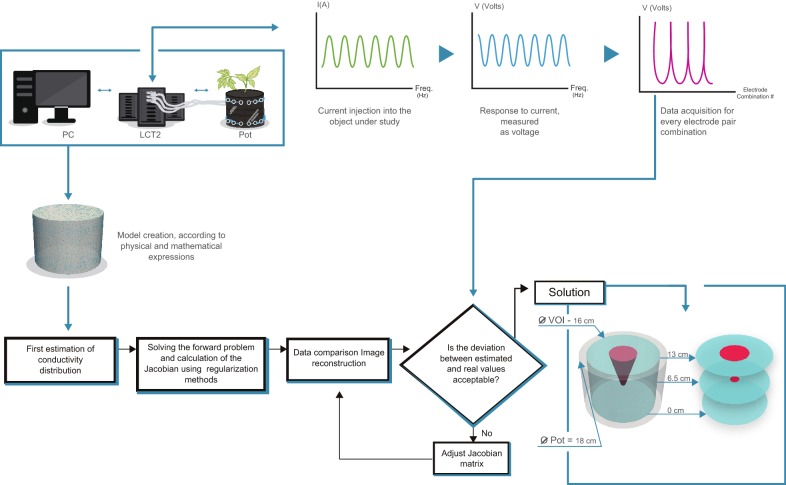
Schematic of signal acquisition and processing for EIT

A flow diagram outlining the key steps for EIT imaging of plant roots is shown in Fig. 8. The alternating current I(A) is introduced via opposite pairs of electrodes and voltage (V) measured at a second pair of electrodes. This is repeated for all electrode combinations. A matrix (‘Jacobian’) representing the voltage sensitivity inside the vessel in response to the injected current is calculated. A theoretical model of the expected conductivity within the pot is created and compared with the experimental values. Initially, the deviation between the theoretical and actual values is large, so values within the Jacobian matrix are adjusted until the deviation is minimised. At this point an acceptable solution has been obtained. This generates a 3-dimensional image reconstruction that describes the impedance of the VOI. One of the prominent effects in the image reconstruction around the electrode locations is ‘ringing’ [56], that is an oscillatory error in the reconstruction due to the step-wise change in the actual conductivity between the electrode material and the medium being measured. Ringing can reduce the resolving power of regions with step-wise conductivity changes, with this effect most prominent near the edges of the vessel. Therefore, the VOI only considers the contents within a 16 cm diameter, avoiding the edges. Furthermore, to ease the analysis of the system, the VOI was divided into upper and lower sections.

Images were reconstructed using MATLAB (Mathworks, Cambridge, UK) and the EIDORS toolkit (v3.8) [57]. The Conjugate Gradient algorithm was selected due to its speed and robustness, enhanced using Tikhonov’s regularisation scheme. The reconstructions were obtained using a 0.05 tolerance and 10 maximum iterations according to Mozorov’s discrepancy principle for CG algorithms. A finite element mesh, required for image reconstruction, was created using Netgen (v5.3) [58], consisting of 16,055 elements, 4906 surface elements, and 4045 points with a mesh quality factor of Q = 0.75. These parameters were selected to provide a medium density mesh ensuring a good trade-off between the accuracy of the solution obtained and the time taken for computation. The initial conductivities used for the reconstruction forward models were in the range of 80–160 mS/cm based on values obtained before seedling transplantation. Data analysis of the 3D reconstructions was performed using ParaView (v5.2).

The average value of each VOI section (whole pot, lower plane, and upper plane) is calculated for each day of the experiment. Since the conductivity changes are primarily negative, we employed their SDs to address the conductivity variation between samples.

## Additional files

**Additional file 1.** Conductivity changes across volume of interests for pots containing soil only. Average values across the (a) VOI and (b) and its standard deviation within the VOI are shown for the entire pot, upper and lower regions. Measurements taken from 3 replicate pots. Results are normalised to Day 0 for each replicate. Axis scales have been set to those in Figure 4.

**Additional file 2.** Root systems of control and infected plants at the end of the experiment.

## Data Availability

The datasets used and/or analysed during the current study are available from the corresponding author on reasonable request.
