# Supplementary figures and images for: Electrical impedance tomography as a tool for phenotyping plant roots
Source: Plant Methods. 2019 May 21;15:49. doi: 10.1186/s13007-019-0438-4 (PMC6528207; doi:10.1186/s13007-019-0438-4)

Entire

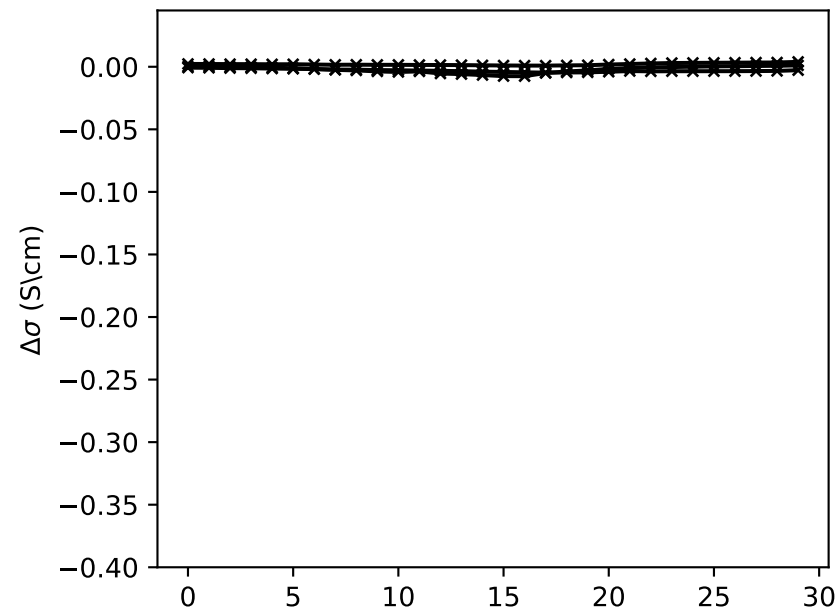

Lower

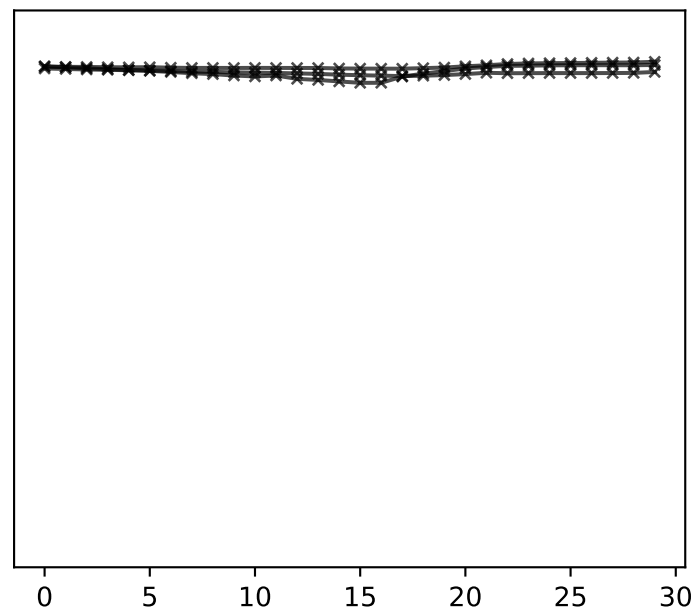

Upper

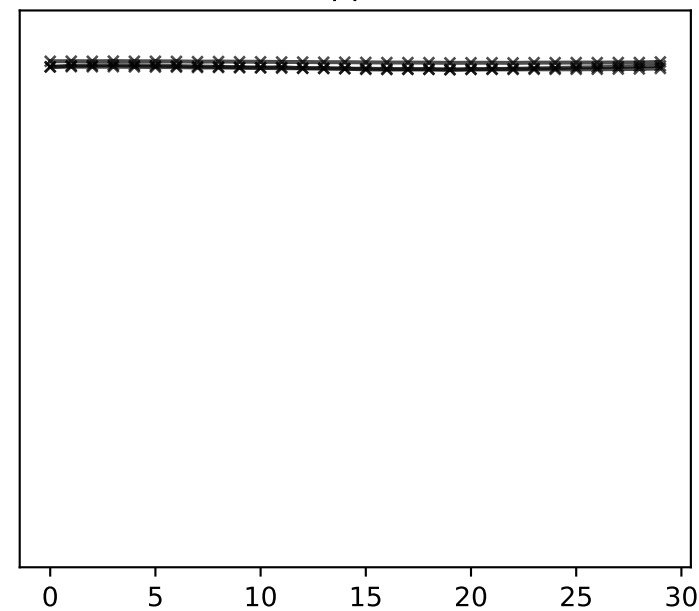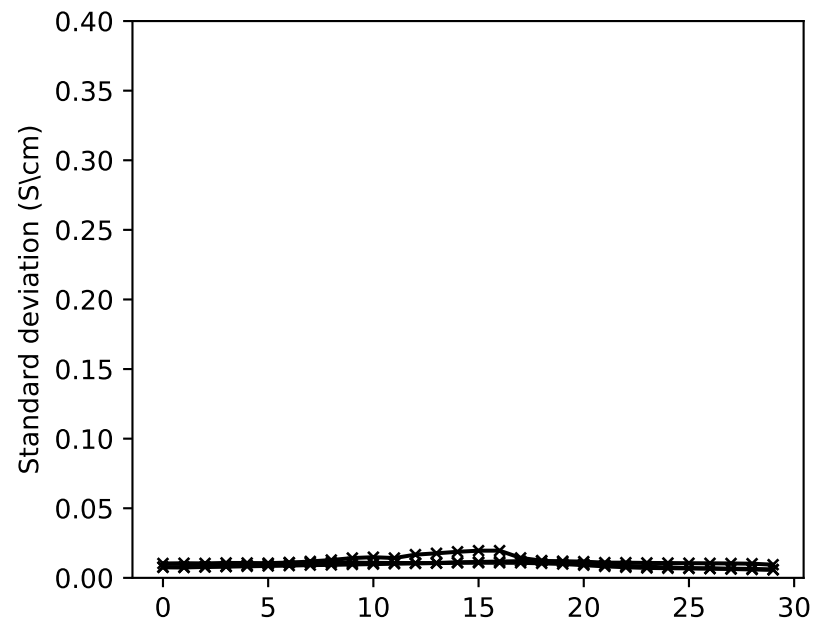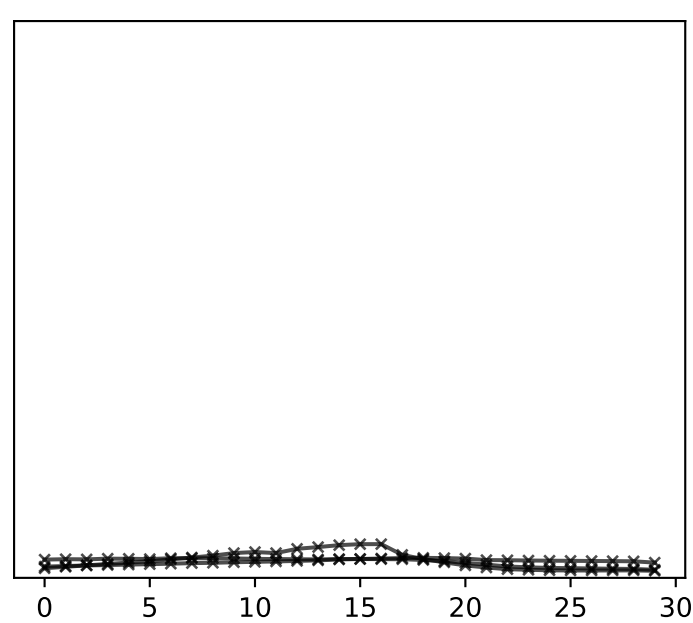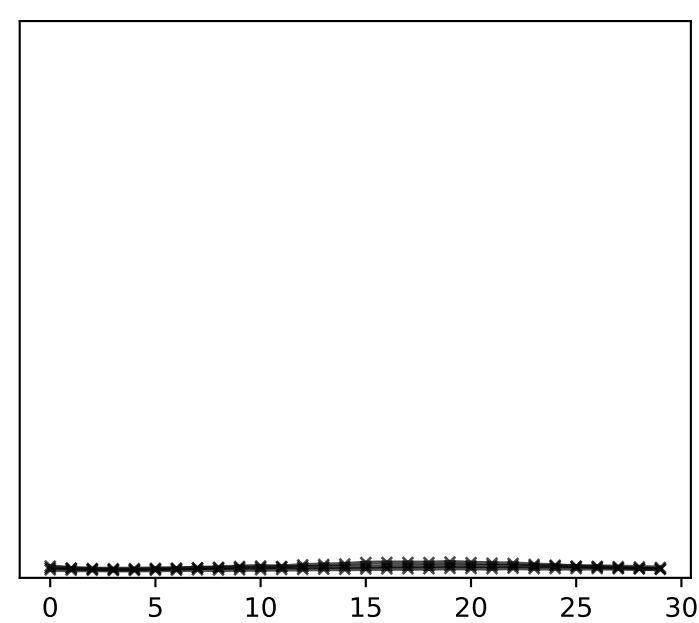

Supplement: Supplementary file 1 — Additional file 1. Conductivity changes across volume of interests for pots containing soil only. Average values across the (a) VOI and (b) and its standard deviation within the VOI are shown for the entire pot, upper and lower regions. Measurements taken from 3 replicate pots. Results are normalised to Day 0 for each replicate. Axis scales have been set to those in Figure 4. [file 13007_2019_438_MOESM1_ESM.pdf]

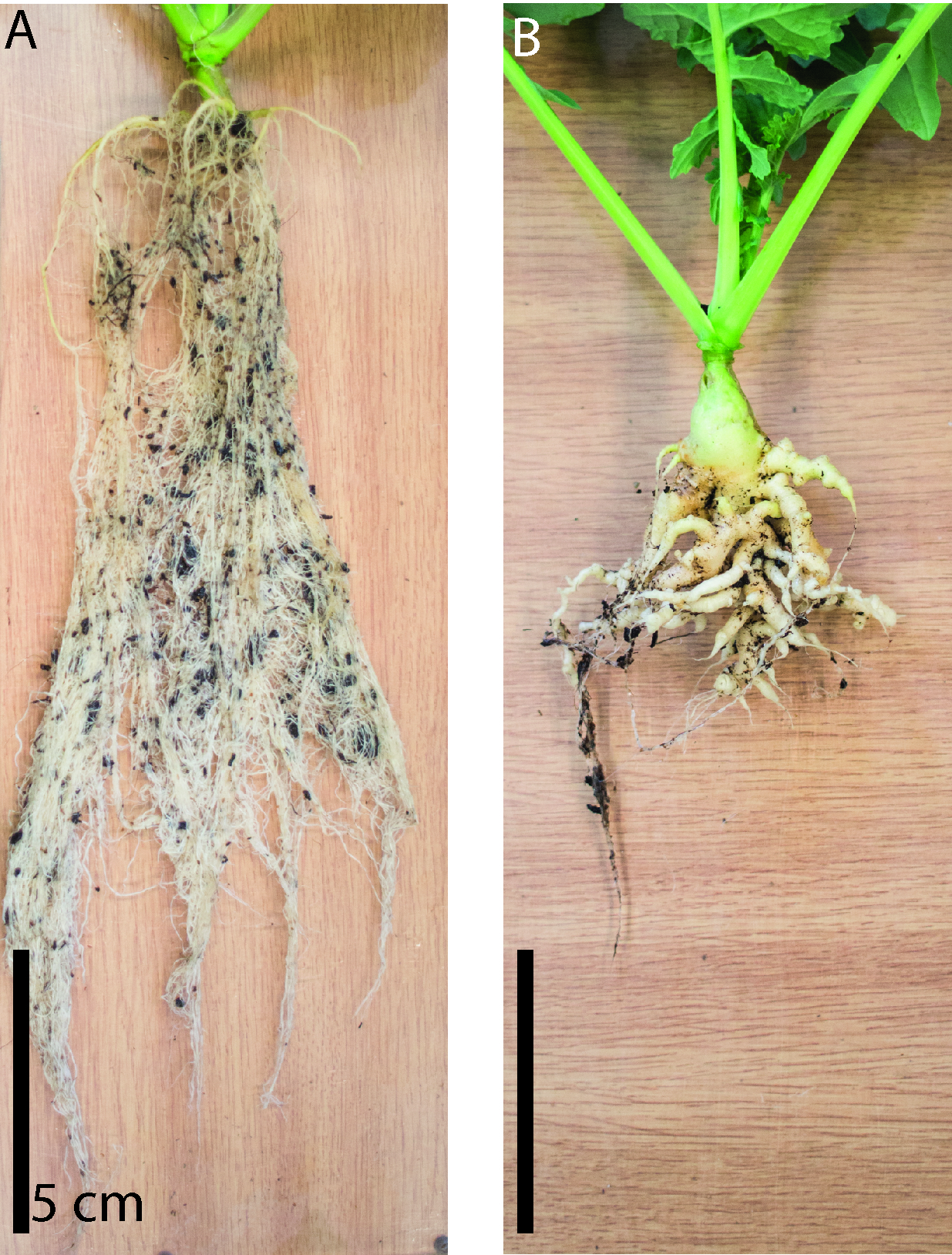

Supplement: Supplementary file 2 — Additional file 2. Root systems of control and infected plants at the end of the experiment. [file 13007_2019_438_MOESM2_ESM.jpg]
